# Deriving stratified effects from joint models investigating gene-environment interactions

**DOI:** 10.1186/s12859-020-03569-4

**Published:** 2020-06-18

**Authors:** Vincent Laville, Timothy Majarian, Paul S. de Vries, Amy R. Bentley, Mary F. Feitosa, Yun J. Sung, D. C. Rao, Alisa Manning, Hugues Aschard, on behalf of the CHARGE Gene-Lifestyle Interactions Working Group

**Affiliations:** 1 Department of Computational Biology, USR 3756 CNRS, Institut Pasteur, Paris, France; 2 Program in Medical and Population Genetics, Broad Institute of MIT and Harvard, Cambridge, MA 02142 USA; 3 Human Genetics Center, Department of Epidemiology, Human Genetics, and Environmental Sciences, School of Public Health, The University of Texas Health Science Center at Houston, Houston, TX 77030 USA; 4 Center for Research on Genomics and Global Health, National Human Genome Research Institute, National Institutes of Health, Bethesda, MD 20892 USA; 5 Division of Biostatistics, Department of Genetics, Washington University School of Medecine, St. Louis, MO 63110 USA; 6 Center for Human Genetics Research, Massachusetts General Hospital, Boston, MA 02114 USA; 7 Program in Genetic Epidemiology and Statistical Genetics, Harvard T.H. Chan School of Public Health, Boston, MA 02115 USA

**Keywords:** Gene-environment interaction, Binary exposure, Stratified analysis, Summary statistics

## Abstract

**Background:**

Models including an interaction term and performing a joint test of SNP and/or interaction effect are often used to discover Gene-Environment (GxE) interactions. When the environmental exposure is a binary variable, analyses from exposure-stratified models which consist of estimating genetic effect in unexposed and exposed individuals separately can be of interest. In large-scale consortia focusing on GxE interactions in which only the joint test has been performed, it may be challenging to get summary statistics from both exposure-stratified and marginal (i.e not accounting for interaction) models.

**Results:**

In this work, we developed a simple framework to estimate summary statistics in each stratum of a binary exposure and in the marginal model using summary statistics from the “joint” model. We performed simulation studies to assess our estimators’ accuracy and examined potential sources of bias, such as correlation between genotype and exposure and differing phenotypic variances within exposure strata. Results from these simulations highlight the high theoretical accuracy of our estimators and yield insights into the impact of potential sources of bias. We then applied our methods to real data and demonstrate our estimators’ retained accuracy after filtering SNPs by sample size to mitigate potential bias.

**Conclusions:**

These analyses demonstrated the accuracy of our method in estimating both stratified and marginal summary statistics from a joint model of gene-environment interaction. In addition to facilitating the interpretation of GxE screenings, this work could be used to guide further functional analyses. We provide a user-friendly Python script to apply this strategy to real datasets. The Python script and documentation are available at https://gitlab.pasteur.fr/statistical-genetics/j2s.

## Background

Gene-Environment (GxE) interactions are of great interest in deciphering biological mechanisms underlying complex human traits and diseases. Several theoretical approaches [1–3] and applications [4–7] have recently been published that identify such GxE interactions. A strategy to detect these interactions applies linear regression models including a GxE interaction term and testing for the hypothesis of null main genetic effect size and GxE interaction effect size, also referred to as the “joint” test [8, 9]. Although several interactions have been associated with different traits using this joint test, the main limitation is that of large sample sizes requirements to reach a suitable statistical power [10]. The Gene-Lifestyle Interaction Working Group is an international, large-scale, multi-ancestry initiative within the Cohorts for Heart and Aging Research in Genomic Epidemiology (CHARGE) consortium that aims to systematically evaluate genome-wide GxE interactions on cardiovascular disease related traits using genotypic data from up to 610,475 individuals [11]. This working group has already unraveled significant GxE interactions using the joint test [12–15]. Nevertheless, in the case of binary exposures, alternative approaches can be of interest, notably to identify differential genetic effects between unexposed and exposed individuals. This strategy requires summary statistics computed in each group of individuals separately, which may not always be available in large-scale consortia. Because of logistical challenges, it can be difficult to obtain these summary statistics in such consortia including tens of individual cohorts.

To benefit from these consortia in which only summary statistics in the joint testing framework may be available, we developed a simple tool to infer summary statistics in the groups of unexposed and exposed individuals separately, as well as summary statistics from the regression model without the GxE interaction term. First, we showed that these summary statistics can be efficiently derived from the joint model assuming independence between genotypes and exposure. We then performed a series of simulations to assess the accuracy of these estimations and to examine the impact of different potential sources of bias. Finally, we applied our pipeline to real data from the Gene-Lifestyle Interactions Working group within the CHARGE Consortium.

## Theoretical derivations

Consider a trait *Y*, a dichotomous exposure *E* and a SNP *G* coded as the number of minor alleles. A framework to test Gene-Environment interactions is based on the generalized linear model:

$$ g\left(\mathbbm{E}\left[Y|G\right]\right)=\alpha +\beta G+\gamma E+\delta G E $$

where *g* denotes either the identity function if *Y* is a quantitative trait or the logit function if *Y* is a binary phenotype.

The marginal model (i.e excluding the interaction term) in unexposed individuals (E = 0), exposed individuals (E = 1) and all individuals are defined as:

$$ {\displaystyle \begin{array}{c}g\left(\mathbbm{E}\left[Y\left|G,E=0\right.\right]\right)={\alpha}_{unexp}+{\beta}_{unexp}G\\ {}g\left(\mathbbm{E}\left[Y\left|G,E=1\right.\right]\right)={\alpha}_{exp}+{\beta}_{exp}G\\ {}g\left(\mathbbm{E}\left[Y|G\right]\right)={\alpha}_{marg}+{\beta}_{marg}G+\gamma E\end{array}} $$

Assuming independence between the genotypes and the exposure (i.e $\mathbbm{E}\left[G\left|E\right.=0\right]=\mathbbm{E}\left[G\left|E\right.=1\right]=G$), the joint model can be used to retrieve the marginal genetic effects *β*_*unexp*_ and *β*_*exp*_ in unexposed (*e* = 0) and exposed (*e* = 1) individuals respectively:

$$ {\displaystyle \begin{array}{c}g\left(\mathbbm{E}\left[Y\left|G,E=e\right.\right]\right)=\alpha +\beta G+\gamma e+\delta G e\\ {}=\alpha +\left(\beta +\delta e\right)G+\gamma e\end{array}} $$

Then setting e to either 0 or 1, marginal effect sizes in unexposed individuals $\hat{\beta_{unexp}}$ and in exposed individuals $\hat{\beta_{exp}}$ can be derived from the genetic and interaction effect sizes ($\hat{\beta}$ and $\hat{\delta}$ respectively) estimated in the joint model:

$$ \hat{\beta_{unexp}}=\hat{\beta},{\sigma}_{\hat{\beta_{unexp}}}={\sigma}_{\hat{\beta}} $$

$$ \hat{\beta_{exp}}=\hat{\beta}+\hat{\delta},{\sigma}_{\hat{\beta_{exp}}}=\sqrt{\sigma_{\hat{\beta}}^2+{\sigma}_{\hat{\delta}}^2+2\mathit{\operatorname{cov}}\left({\sigma}_{\hat{\beta}},{\sigma}_{\hat{\delta}}\right)} $$

where ${\sigma}_{\hat{\beta}}$ and ${\sigma}_{\hat{\delta}}$ denote respectively the standard errors of the genetic effect and interaction effect in the joint model.

Similarly, summary statistics in the marginal model (excluding the interaction term) can be derived from the joint model:

$$ {\displaystyle \begin{array}{c}g\left(\mathbbm{E}\left[Y|G\right]\right)=g\left(\mathbbm{E}\left[Y\left|E=0\right.\right]\right)\times \mathbb{P}\left(E=0\right)+g\left(\mathbbm{E}\left[Y\left|E=1\right.\right]\right)\times \mathbb{P}\left(E=1\right)\\ {}=\left[\ \alpha +\beta G\right]\times \left(1-{\mu}_E\right)+\left[\ \alpha +\beta G+\gamma +\delta G\right]\times {\mu}_E\\ {}=\left(\alpha +\gamma {\mu}_E\right)+\left(\beta +\delta {\mu}_E\right)G\end{array}} $$

Hence, the marginal genetic effect $\hat{\beta_{marg}}$ and its standard error ${\sigma}_{\hat{\beta_{marg}}}$ are equal to:

$$ {\displaystyle \begin{array}{c}\hat{\beta_{marg}}=\hat{\beta}+\hat{\delta}{\mu}_E\\ {}{\sigma}_{\hat{\beta_{marg}}}=\sqrt{\sigma_{\hat{\beta}}^2+{\mu}_E^2{\sigma}_{\hat{\delta}}^2+2{\mu}_E\mathit{\operatorname{cov}}\left({\sigma}_{\hat{\beta}},{\sigma}_{\hat{\delta}}\right)}\end{array}} $$

## Implementation

We developed a Python script to derive summary statistics in the marginal model and in each group of individuals separately. As input, the script takes one file with the summary statistics from the joint model, that are genetic and interaction effect sizes, their standard errors, the correlation between the two effect sizes and the sample size per SNP corresponding to the number of genotypes available for this SNP (which may differ from the sample size of the study because of missing data). This file corresponds to the output of the METAL software to meta-analyze GxE screenings using the joint test [9]. In addition to this file, the script also takes two arguments that are the total sample size *N* of the study and the number of exposed individuals *N*_*e*_ included in the study. These two arguments are used to infer the sample sizes $$ {N}_v\times \raisebox{1ex}{$\left(N-{N}_e\right)$}\!\left/ \!\raisebox{-1ex}{$N$}\right. $$ and $$ {N}_v\times \raisebox{1ex}{${N}_e$}\!\left/ \!\raisebox{-1ex}{$N$}\right. $$ in the group of unexposed and exposed individuals respectively for each SNP, where *N*_*v*_ is the sample size for the SNP. We also implemented a filtering procedure to exclude variants with a low sample size compared to the distribution of the sample sizes: a SNP with a sample size below the 9th decile of the sample size distribution divided by 1.5 is excluded from the analysis. As output, the script generates a single file containing the genetic effect size and its standard error in the group of unexposed individuals, in the group of exposed individuals and in the total sample. The script and a detailed documentation using an example are available at https://gitlab.pasteur.fr/statistical-genetics/j2s.

## Results

### Simulation study

**Fig. 1:**
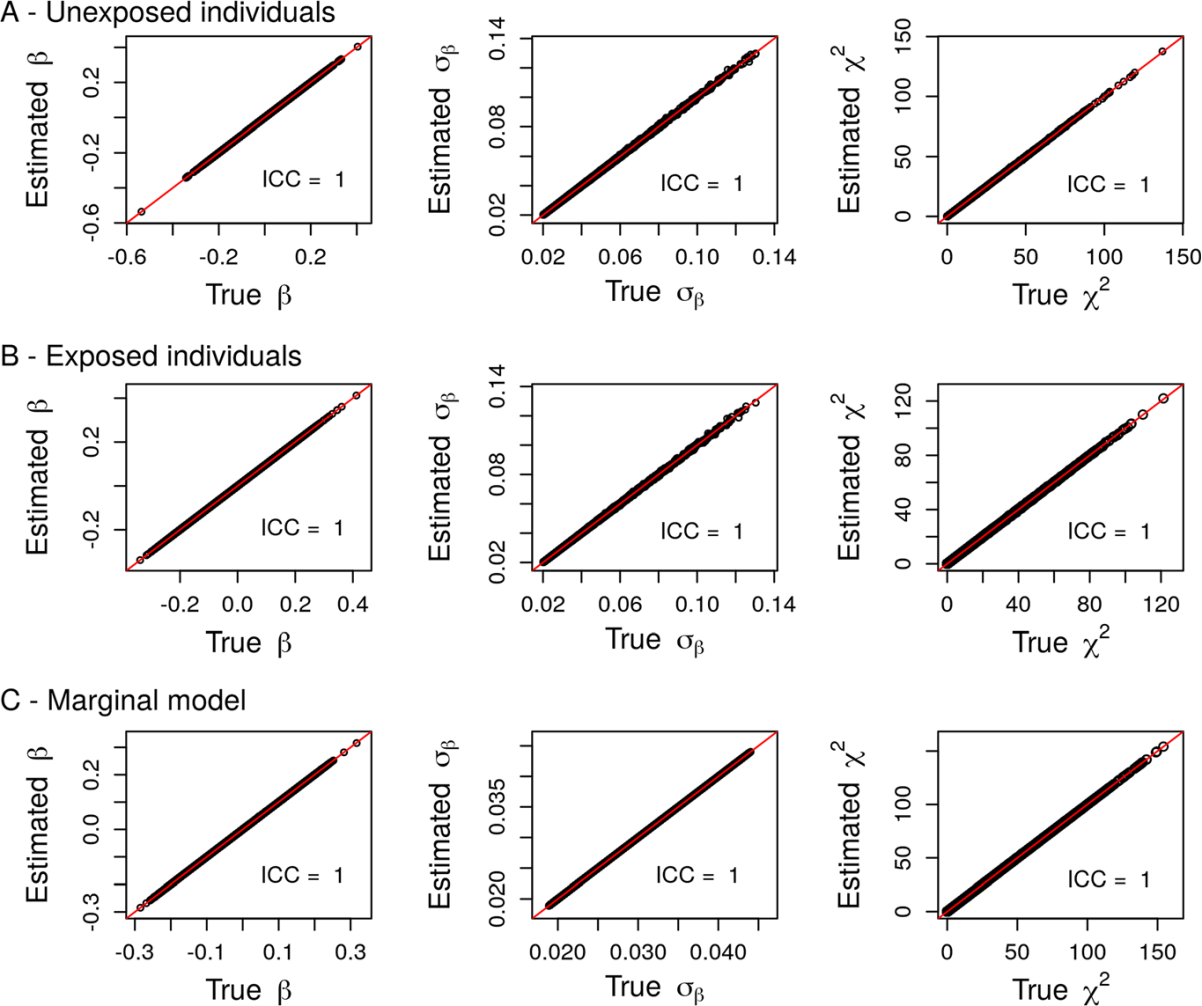
Comparison between summary statistics derived from individual-level data (True) and their estimations (Estimated) in unexposed (**a**) and exposed (**b**) individuals and in the marginal model (**c**) using simulated data in the case of a quantitative phenotype

First, we performed a simulation study to assess the accuracy of the estimations obtained from the theoretical results described above. In each of the 1000 replicates, we simulated 10,000 genotypes of a SNP with a random MAF between 1 and 50% and a binary exposure with a random probability of being exposed ranging from 0.1 to 0.5. Then, we simulated a continuous phenotype *Y* = *β*_*G*_*G* + *β*_*G*_*E* + *β*_*GE*_*G* × *E* + *ε* as a linear combination of the SNP *G*, the exposure *E* and the *G* × *E* interaction term with randomly chosen effect sizes *β*_*G*_, *β*_*E*_ and *β*_*GE*_ and a random noise $\varepsilon \sim \mathcal{N}\left(0,{\sigma}^2\right)$. The effect sizes *β*_*G*_, *β*_*E*_ and *β*_*GE*_ were drawn from a uniform distribution on [0.05; 0.2] with a randomly and equiprobably chosen sign. Note that in this design, genotypes *G* and exposure *E* were drawn independently. Then, on the one hand, we computed the summary statistics from the joint model including the GxE interaction term using individual level data. On the other hand, we applied linear regressions without the GxE interaction term in each group of individuals (unexposed and exposed) separately and in the pooled sample to compute the summary statistics of the genetic effect in each group of individuals and in the marginal model. Using the estimators derived from the joint model, we also inferred these summary statistics in each group and in the marginal model using our pipeline. Comparisons of the empirical and inferred summary statistics showed high accuracy of the estimators, with intraclass correlation coefficient (ICC) between “real” and “estimated” equal to 1 in all scenarios (Fig. 1).

We also performed this simulation study for a binary trait. For each of the 10,000 replicates, we generated random effect sizes *β*_*G*_, *β*_*E*_ and *β*_*GE*_ as described above and then simulated a binary outcome from a Bernouilli distribution, with the probability of being a case as a binary trait from using a logistic model as $$ P\left(Y=1\right)=\raisebox{1ex}{$1$}\!\left/ \!\raisebox{-1ex}{$\left(1+{e}^{\alpha +{\beta}_GG+{\beta}_GE+{\beta}_{GE}G\times E+\varepsilon}\right)$}\right. $$, where $$ \varepsilon \sim \mathcal{N}\left(0,{\sigma}^2\right) $$. We then conducted the same analyses as for quantitative traits by performing logistic regressions instead of linear regressions to compare the summary obtained using individual-level data to those estimated by our pipeline. As for quantitative traits, the estimator was highly accurate (Figure S1).

### Potential bias sources

We performed several complementary simulation studies to assess the contribution of several bias sources. Each time, we generated genotypes for 50,000 individuals and repeated the analysis 10,000 times.

**Fig. 2:**
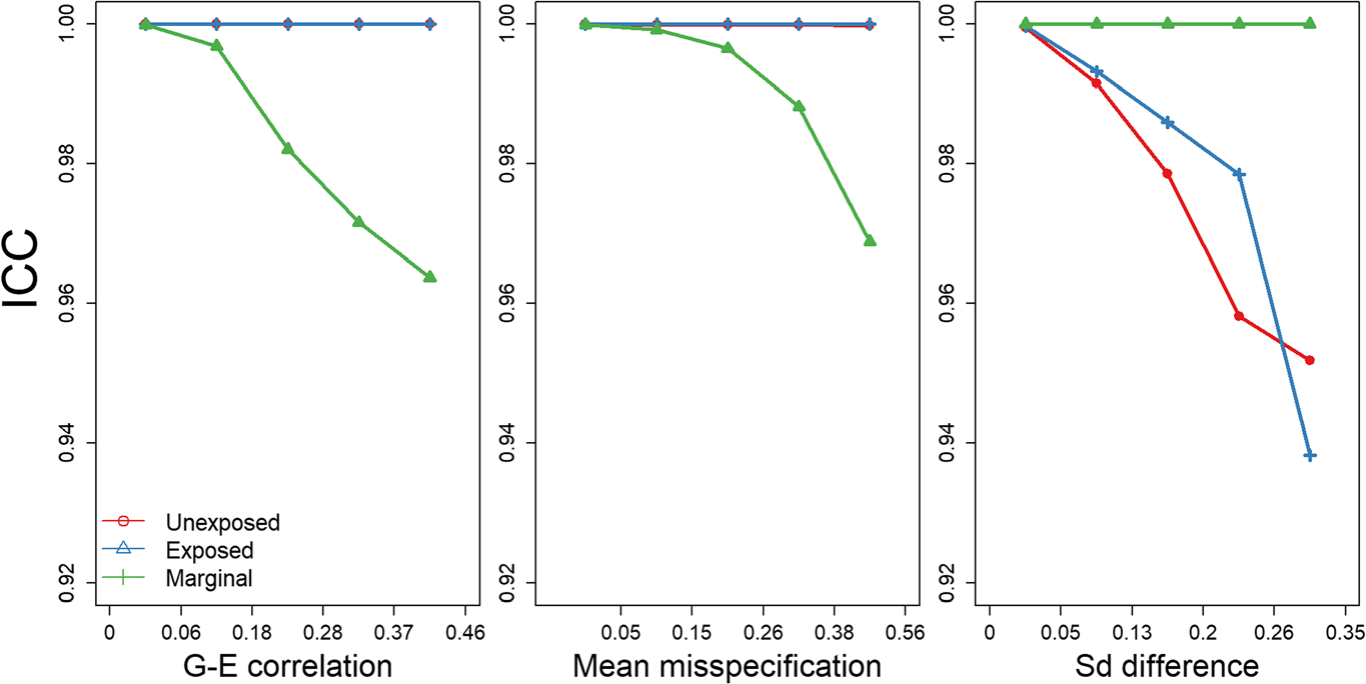
Impact of the different sources of bias on the estimations. The Intraclass Correlation Coefficient (ICC) between the test statistics from real data analysis and the test statistics estimated from the summary statistics in the joint model in unexposed individuals only (red), exposed individuals only (blue) and in the marginal model (green) are plotted by quintiles of the G-E correlation coefficient distribution (left), the difference between the true and estimated proportion of exposed individuals (middle) and the distribution of the difference in phenotypic standard deviation between unexposed and exposed individuals (right)

First, the estimators’ derivation relies on the assumption that genotypes and environment are statistically independent. We performed a simulation study in which correlation existed between genotypes and the environment. We then compared our summary statistics estimated from the joint model to summary statistics derived using individual-level data (Figs. 2, S2). Relaxing the G-E independence assumption did not impact the estimator’s accuracy when deriving stratified summary. However, estimations in the marginal model were slightly impacted by the correlation between G and E. Indeed, inferred effect sizes are a little biased and effect sizes standard errors are overestimated. Although estimation errors increase with the correlation, the impact on the test statistics remains very limited.

Second, bias can occur because of a misspecification of the proportion of exposed individuals. This is very likely to happen for SNPs with a low sample size (because of missing genotypes) compared to the maximum sample size. To evaluate the impact of such a misspecification, for each SNP, we selected a subset of individuals to include only a randomly selected proportion of individuals while intentionnaly misspecifying the proportion of exposed individuals. For each subset, we removed a randomly selected number of exposed individuals. We the compared the summary statistics obtained using the individual-level data and those estimated using the pipeline. As expected from the theoretical derivations detailed above, misspecifying the proportion of exposed individuals, quantified as $$ \raisebox{1ex}{$\left|{\mu}_E-{m}_E\right|$}\!\left/ \!\raisebox{-1ex}{${\mu}_E$}\right. $$ where *μ*_*E*_ is the mean of the exposure in the whole sample and *m*_*E*_ is the mean of the exposure in the subsample, only impacted estimations in the marginal model including all individuals. Notably, the larger the difference between the true (in the subset of selected individuals) and the estimated (computed in the whole sample) proportions of exposed individuals, the larger are the discrepancies between the summary statistics (Figs. 2, S3).

Third, bias in our estimations can also occur due to differences in phenotypic variance between unexposed and exposed individuals. To explore this, we simulated a phenotype with exposure-dependent variance by adding statistical noise to the phenotypes of exposed individuals and performed the same simulation study as described above. A different phenotypic variance in the two groups of individuals did not bias estimation of the summary statistics in the marginal model but it clearly biased the estimation of summary statistics in the exposed and unexposed individuals (Figs. 2, S4). Although this exposure-dependent phenotypic variance did not impact the estimation of the effect sizes, it biased the estimation of the effect size standard error. Standard errors tend to be overestimated in the group in which the phenotypic variance is the largest, leading to deflated test statistics and conversely. Importantly, the larger differences in phenotypic variance yielded larger induced biases.

Finally, we also generated data (50,000 individuals and 10,000 iterations) under a null model with neither a genetic effect nor an interaction effect to assess the control of the type I error rate and quantify the discrepancies in significance results that can arise because of these different sources of bias (Figures S5, S6). Globally, the type I error rate is well-controlled in the presence of G-E correlation and for SNPs with low sample compared to the total sample size (Figure S5), but the systematic inflation (resp. deflation) of chi-square statistics observed in a group of individuals when the phenotypic variance differs depending on the exposure (Figure S4) leads to an uncontrolled type I error rate (Figure S5). However, important discrepancies in the significance assessment evaluated as the proportion of SNPs significant using Bonferroni-adjusted *p*-values with only one of the two methods (using individuals-level data or the estimation pipeline) can be observed, confirming the impact of these source of bias (Figure S6).

### Real data application

**Fig. 3:**
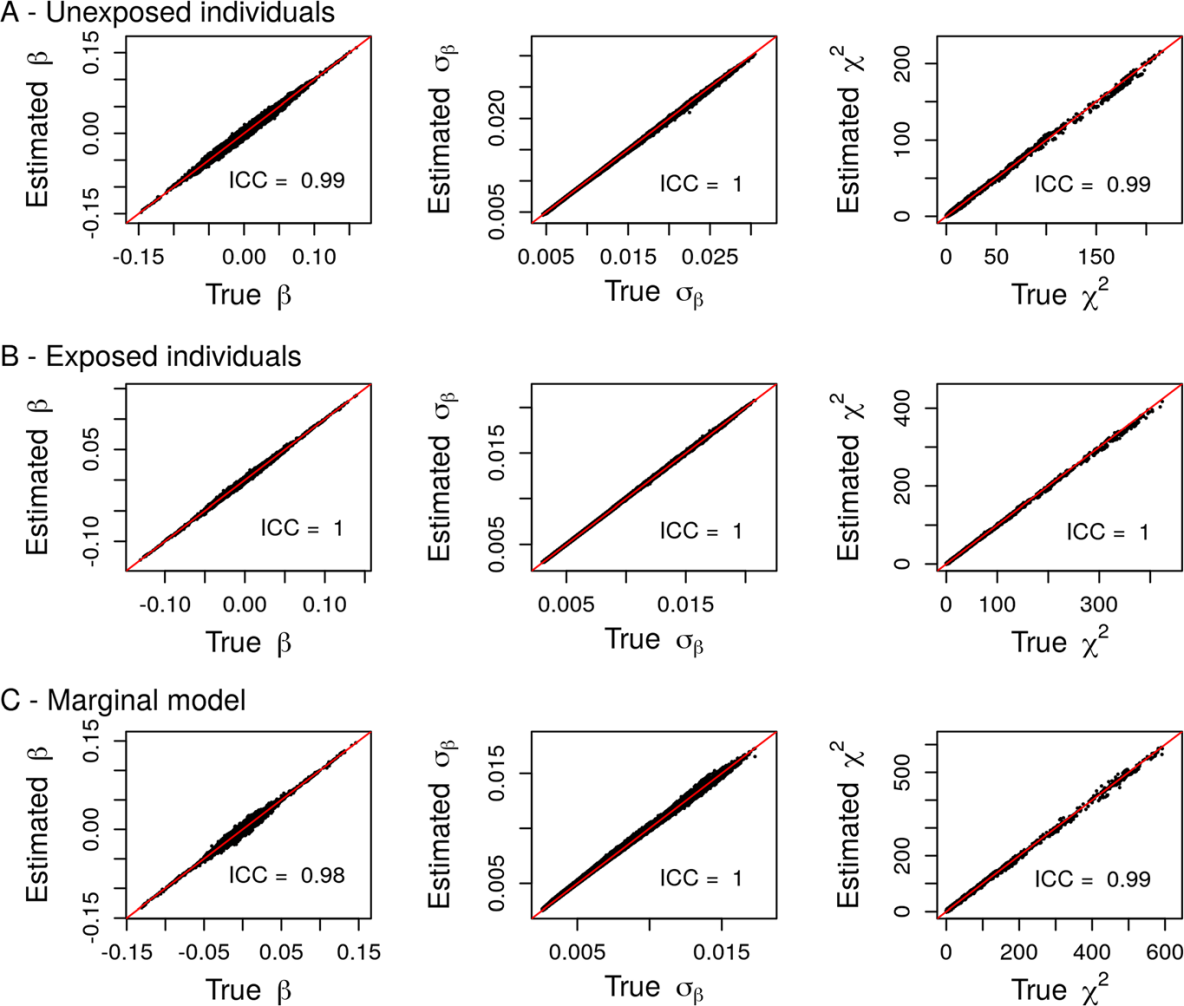
Comparison between summary statistics derived from individual-level data (True) and their estimations (Estimated) in unexposed (**a**) and exposed (**b**) individuals and in the marginal model (**c**) using real data summary statistics from the SNP by alcohol screenings on triglycerides

**Fig. 4:**
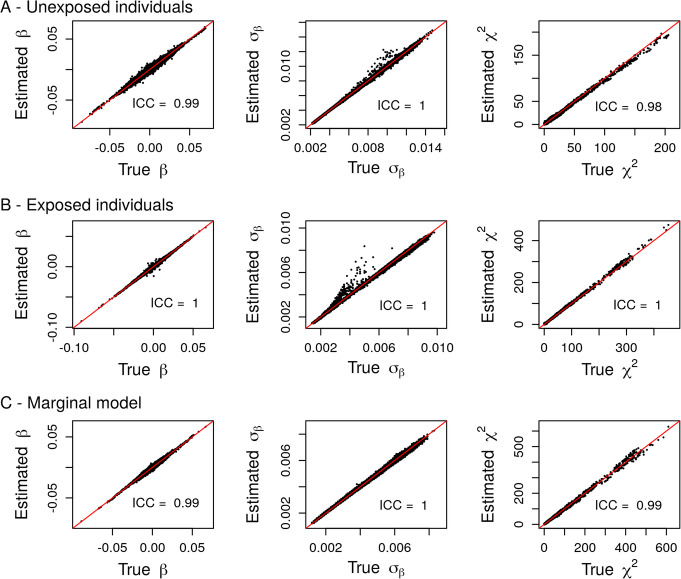
Comparison between summary statistics derived from individual-level data (True) and their estimations (Estimated) in unexposed (**a**) and exposed (**b**) individuals and in the marginal model (**c**) using real data summary statistics from the SNP by alcohol screenings on High Density Lipoproteins

**Fig. 5:**
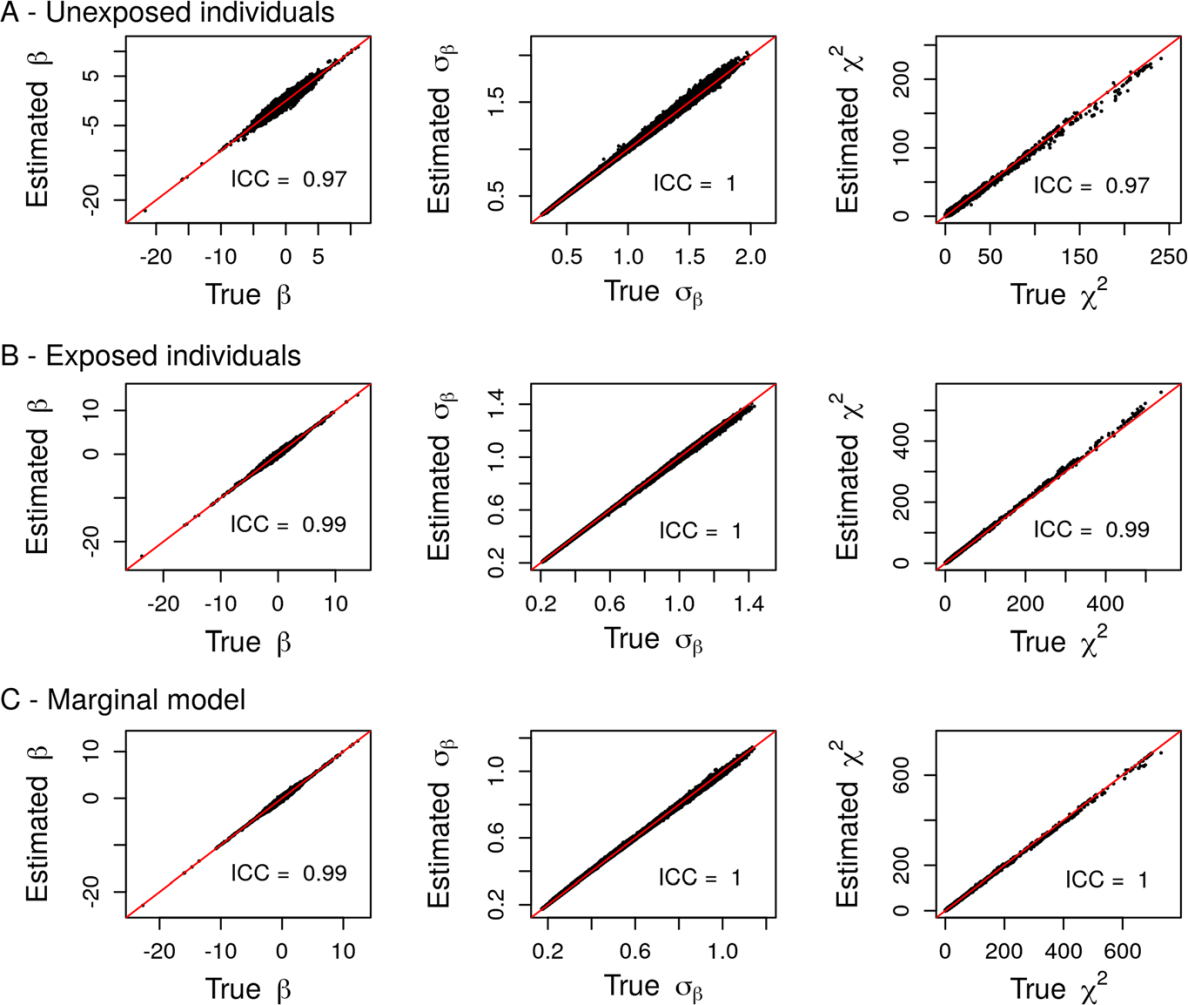
Comparison between summary statistics derived from individual-level data (True) and their estimations (Estimated) in unexposed (**a**) and exposed (**b**) individuals and in the marginal model (**c**) using real data summary statistics from the SNP by alcohol screenings on Low Density Lipoproteins

We assessed the accuracy of our estimations using real data from the Gene-Lifestyle Interaction Working Group of the CHARGE consortium [11]. This Working Group recently published genome-wide SNP-by-alcohol interaction screenings [13] using joint tests and focusing on three lipids level: triglycerides (TG), high-density lipoproteins (HDL), and low-density lipoproteins (LDL). Genome-wide screenings for genetic marginal effects were also performed in unexposed and exposed individuals separately and in the whole sample. Here, we used summary statistics from the genome wide SNP by exposure interaction screenings in individuals from European ancestry and derived marginal summary statistics in unexposed and exposed individuals separately, and in the whole sample. We then compared the inferred summary statistics with the empirical summary statistics derived using individual-level data (Figs. 3, 4, 5). The estimations exhibited high accuracy as demonstrated by the very high ICC between the estimated and true summary statistics (mean ICC = 0.99). Note that some discrepancies are observed for only a very limited number of SNPs (less than 100 out of more than 7 million variants) and do not influence much the ICC, which measures the “agreement” between the true and estimated parameters. Overall, filtering to exclude SNPs with low relative sample size (i.e below the 9th decile of the sample size distribution divided by 1.5) lead to more accurate estimations

## Discussion

In this work, we aimed at inferring marginal genetic effects in exposed and unexposed individuals separately and in the whole sample using summary statistics of the joint test performed in the context of GxE interaction studies. We analytically derived estimators of marginal genetic effects in the different groups of individuals and in the total sample. We validated the method through simulation studies and real data applications which both highlighted the accuracy of our estimations. Notably, this method can be applied without loss of accuracy to quantitative and binary traits.

As demonstrated by our simulation studies, differences between true and estimated parameters are observed for SNPs with the lowest sample sizes. This is explained by a different proportion of exposed individuals for this particular SNP and in the whole sample. Also, our method also provides basic estimates of the expected sample size in the groups of exposed and unexposed individuals. For the same reason, these estimates could be biased for SNPs with low sample size compared to the total sample size. Consequently, we implemented a procedure to filter out variants with low relative sample size to minimize this potential bias.

Our estimations rely on the independence between genotypes and exposures. Relaxing this assumption leads to biased estimations of the marginal effect size standard deviation in the marginal model, but does not impact the accuracy of the estimations in the stratified models. As correlation between SNPs and the exposure cannot be retrieved using summary statistics from the joint model, although this assumption may not hold only for a very limited number of SNPs,existing litterature may be helpful to identify variants which should be discarded from the analyses because of existing correlation with the considered exposure. The correlations between genotypes and exposures are expected to be low, resulting in little overall impact, as observed when validating our estimators using real data from the Gene-Lifestyle Interaction Working Group.

Finally, we evaluated our estimations in the case of exposure-dependent phenotypic variance. Although our simulations showed clear impacts on the estimations in the stratified models, we noted that the error increased with the magnitude of this difference. In real data applications, such differences in phenotypic variance are expected to be small and should consequently have only a limited impact on the estimations in each exposure stratum. Application to real data sets confirmed this notion as our estimations were highly concordant with real data.

Overall, an advantage of exposure-stratified models is that they allow for a comparison between genetic effects in each group of individuals. This different way of quantifying GxE interactions makes the interpretation more intuitive compared to the joint test by comparing genetic effects between the two groups. In addition, exposure-stratified summary statistics can also be used to apply further analyses such as biological pathways [16] or heritability-based [17–19] analyses. Results from those analyses in each group could then be compared and help better understanding the genetic architecture of the trait. These strategies could also highlight different genetic mechanisms induced by the exposure, opening new path towards public health prevention policies or the identification of potential drug targets.

## Conclusion

In this work, we derived accurate estimations of the marginal genetic effects in unexposed and exposed individuals separately and in the whole sample in the context of genome-wide GxE interaction screenings using the joint test. This method can not only lead to a more intuitive understanding of GxE interactions but also be used to perform additional studies that can guide further functional analyses. We implemented j2s, a Python3 script to easily apply this method, available at https://gitlab.pasteur.fr/statistical-genetics/j2s.

## Availability and requirements

**Project name:** j2s

**Project home page:** https://gitlab.pasteur.fr/statistical-genetics/j2s

**Operating systems:** Linux

**Programming language:** Python3

**Other requirements:** None

**License:** MIT

**Any restrictions to use by non-academics:** None

## Supplementary information

**Additional file 1: Figure S1.** Comparison between summary statistics derived from individual-level data (True) and their estimations (Estimated) in unexposed (A) and exposed (B) individuals and in the marginal model (C) using simulated data in the case of a binary phenotype. **Figure S2.** Comparison between summary statistics derived from individual-level data (True) and their estimations (Estimated) in unexposed (A) and exposed (B) individuals and in the marginal model (C) using simulated data in the case of a quantitative phenotype when relaxing the genotype-environment independence. **Figure S3.** Comparison between summary statistics derived from individual-level data (True) and their estimations (Estimated) in unexposed (A) and exposed (B) individuals and in the marginal model (C) using simulated data in the case of differences between the proportion of exposed individuals for the SNP and the proportion of exposed individuals in the whole sample. **Figure S4.** Comparison between summary statistics derived from individual-level data (True) and their estimations (Estimated) in unexposed (A) and exposed (B) individuals and in the marginal model (C) using simulated data in the case of different phenotypic variance conditionally on the exposition. **Figure S5.** Comparison of the Type I error rate evaluated between summary obtained using individual-level data (blue) and summary statistics estimated using the pipeline (orange) with respect to the different quintiles of the different sources of bias: G-E correlation(A), misspecification of the proportion of exposed individuals (B) and different phenotypic variance in the strata of the exposure (C). Type I error rate were evaluated for the marginal model in all individuals (left), in unexposed individuals only (middle) and in exposed individuals only (right). The dashed line represents the nominal significance threshold (5%). **Figure S6.** Proportion of SNPs with discordant significance results between summary statistics obtained using individual-level data and summary statistics estimated using our pipeline with respect to the different quintiles of the different sources of bias: G-E correlation(A), misspecification of the proportion of exposed individuals (B) and different phenotypic variance in the strata of the exposure (C). Type I error rate were evaluated for the marginal model in all individuals (left), in unexposed individuals only (middle) and in exposed individuals only (right).

## Data Availability

The data that support the findings of this study are part of the The Cohorts for Heart and Aging Research in Genomic Epidemiology (CHARGE) consortium and are available from the authors upon reasonable request.

## Abbreviations

GLIWG: Gene-Lifestyle Interaction Working Group
GxE: Gene-Environment
ICC: Intraclass Correlation Coefficient
SNP: Single Nucleotide Polymorphism
