# Supplementary material for: Deriving stratified effects from joint models investigating gene-environment interactions
Source: BMC Bioinformatics. 2020 Jun 18;21:251. doi: 10.1186/s12859-020-03569-4 (PMC7302007; doi:10.1186/s12859-020-03569-4)
Supplement: Supplementary file 1 — Additional file 1: Figure S1. Comparison between summary statistics derived from individual-level data (True) and their estimations (Estimated) in unexposed (A) and exposed (B) individuals and in the marginal model (C) using simulated data in the case of a binary phenotype. Figure S2. Comparison between summary statistics derived from individual-level data (True) and their estimations (Estimated) in unexposed (A) and exposed (B) individuals and in the marginal model (C) using simulated data in the case of a quantitative phenotype when relaxing the genotype-environment independence. Figure S3. Comparison between summary statistics derived from individual-level data (True) and their estimations (Estimated) in unexposed (A) and exposed (B) individuals and in the marginal model (C) using simulated data in the case of differences between the proportion of exposed individuals for the SNP and the proportion of exposed individuals in the whole sample. Figure S4. Comparison between summary statistics derived from individual-level data (True) and their estimations (Estimated) in unexposed (A) and exposed (B) individuals and in the marginal model (C) using simulated data in the case of different phenotypic variance conditionally on the exposition. Figure S5. Comparison of the Type I error rate evaluated between summary obtained using individual-level data (blue) and summary statistics estimated using the pipeline (orange) with respect to the different quintiles of the different sources of bias: G-E correlation(A), misspecification of the proportion of exposed individuals (B) and different phenotypic variance in the strata of the exposure (C). Type I error rate were evaluated for the marginal model in all individuals (left), in unexposed individuals only (middle) and in exposed individuals only (right). The dashed line represents the nominal significance threshold (5%). Figure S6. Proportion of SNPs with discordant significance results between summary statistics obtained using [file 12859_2020_3569_MOESM1_ESM.docx]

**Stratifying summary statistics from Gene-Environment screenings – Supplementary material**

[Figure S1. 2](#_Toc36228045)

[Figure S2. 3](#_Toc36228046)

[Figure S3 4](#_Toc36228047)

[Figure S4. 5](#_Toc36228048)

[Figure S5. 6](#_Toc36228049)

[Figure S6. 7](#_Toc36228050)

Figure S1. Comparison between summary statistics derived from individual-level data (True) and their estimations (Estimated) in unexposed (A) and exposed (B) individuals and in the marginal model (C) using simulated data in the case of a binary phenotype.


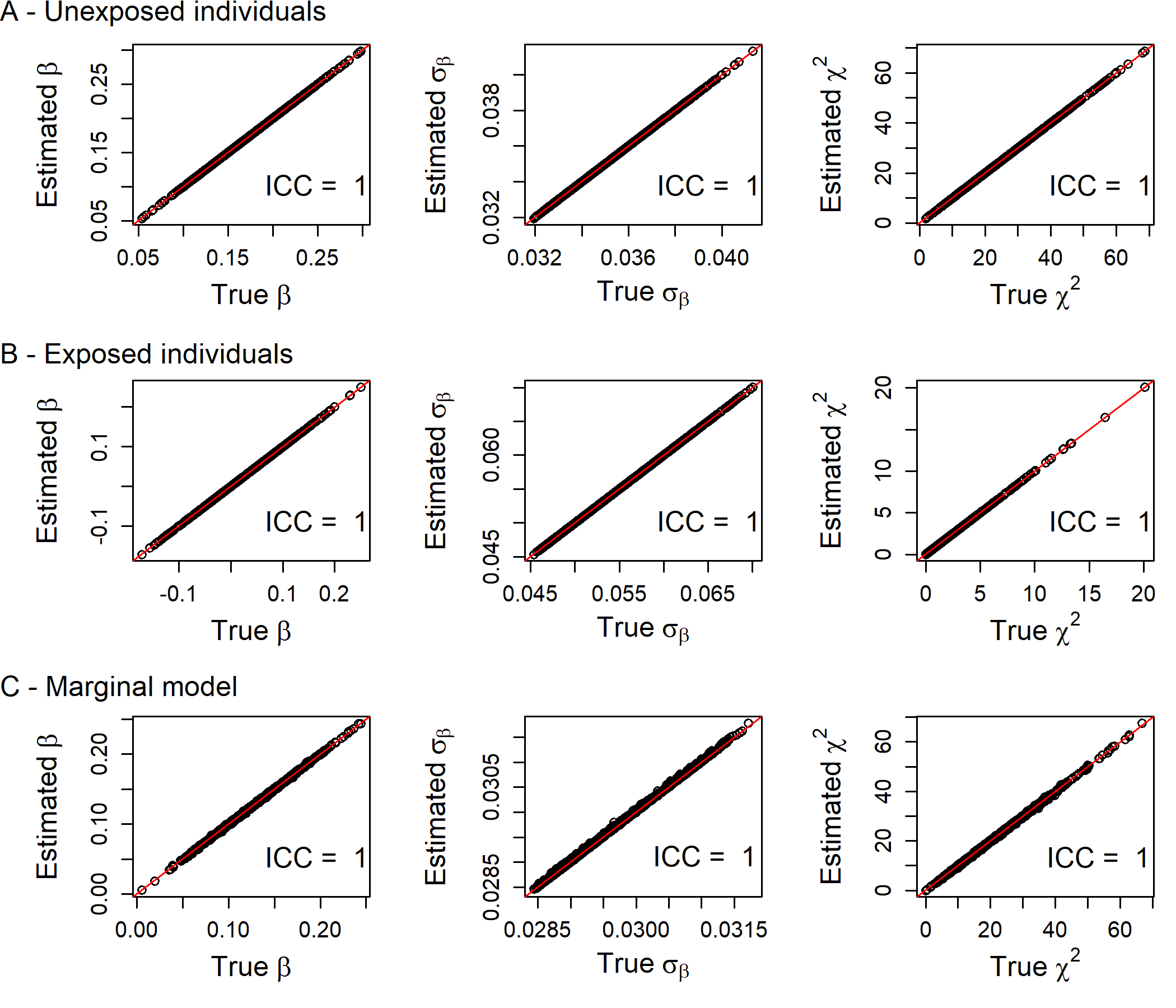


Figure S2. Comparison between summary statistics derived from individual-level data (True) and their estimations (Estimated) in unexposed (A) and exposed (B) individuals and in the marginal model (C) using simulated data in the case of a quantitative phenotype when relaxing the genotype-environment independence.


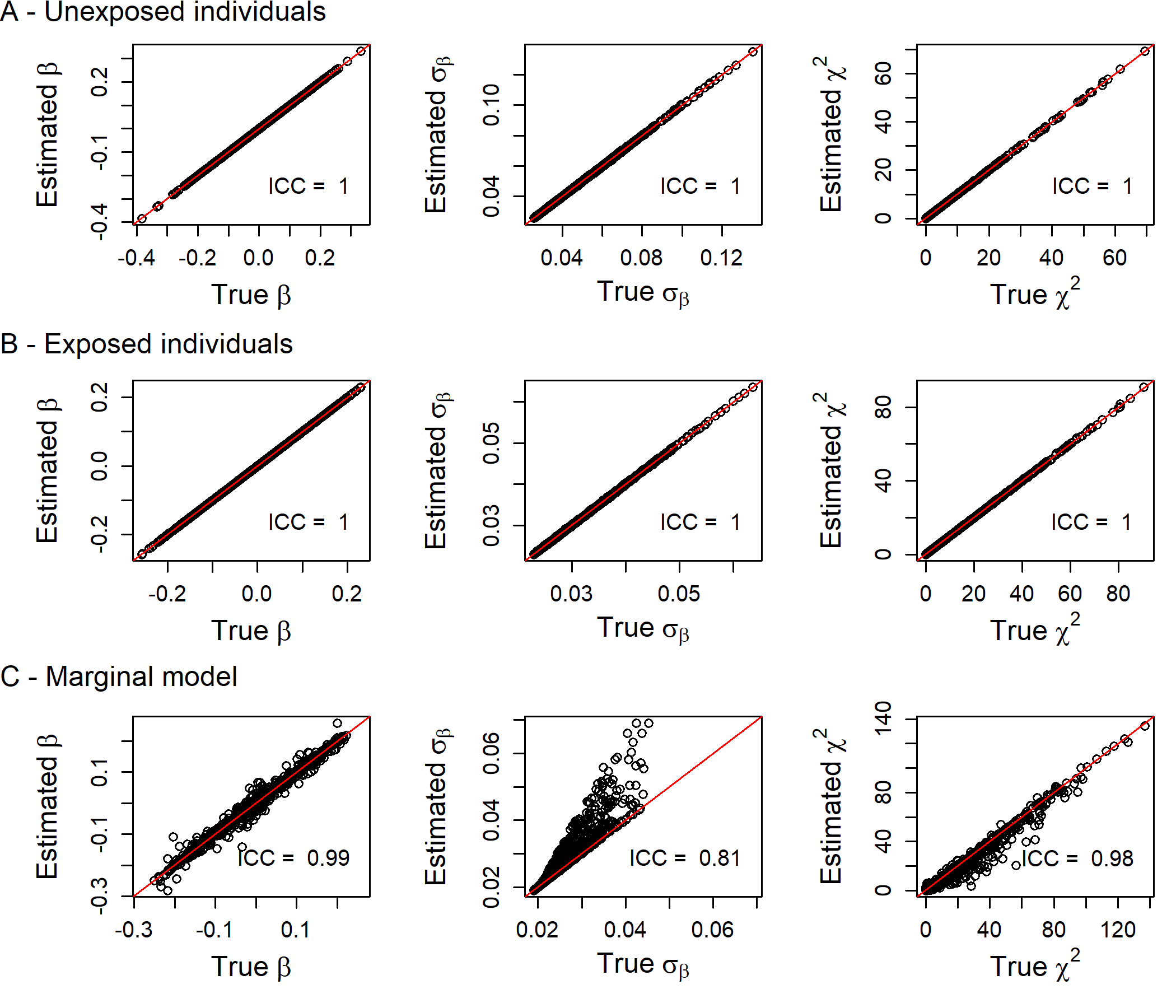


Figure S3. Comparison between summary statistics derived from individual-level data (True) and their estimations (Estimated) in unexposed (A) and exposed (B) individuals and in the marginal model (C) using simulated data in the case of differences between the proportion of exposed individuals for the SNP and the proportion of exposed individuals in the whole sample.


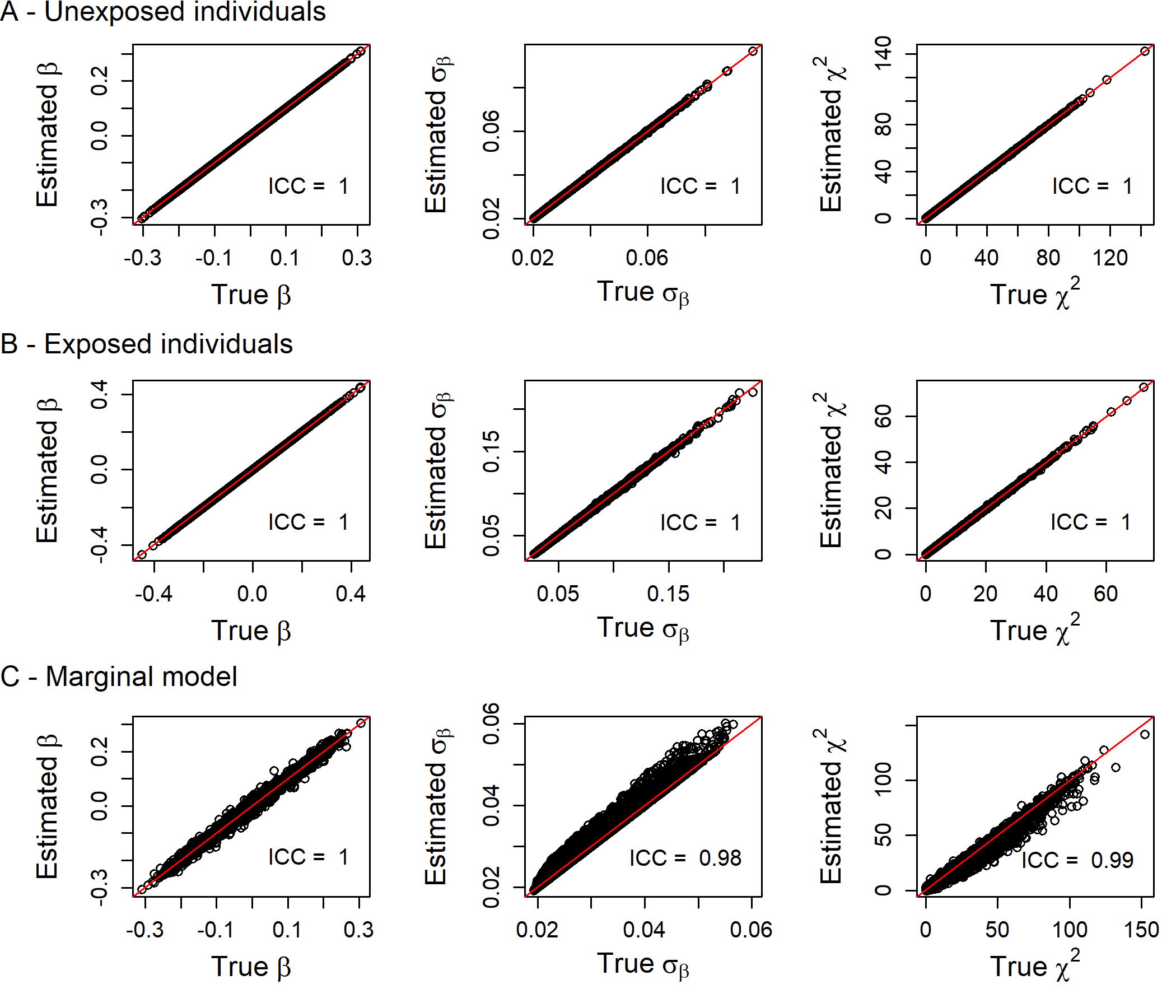


Figure S4. Comparison between summary statistics derived from individual-level data (True) and their estimations (Estimated) in unexposed (A) and exposed (B) individuals and in the marginal model (C) using simulated data in the case of different phenotypic variance conditionally on the exposition.


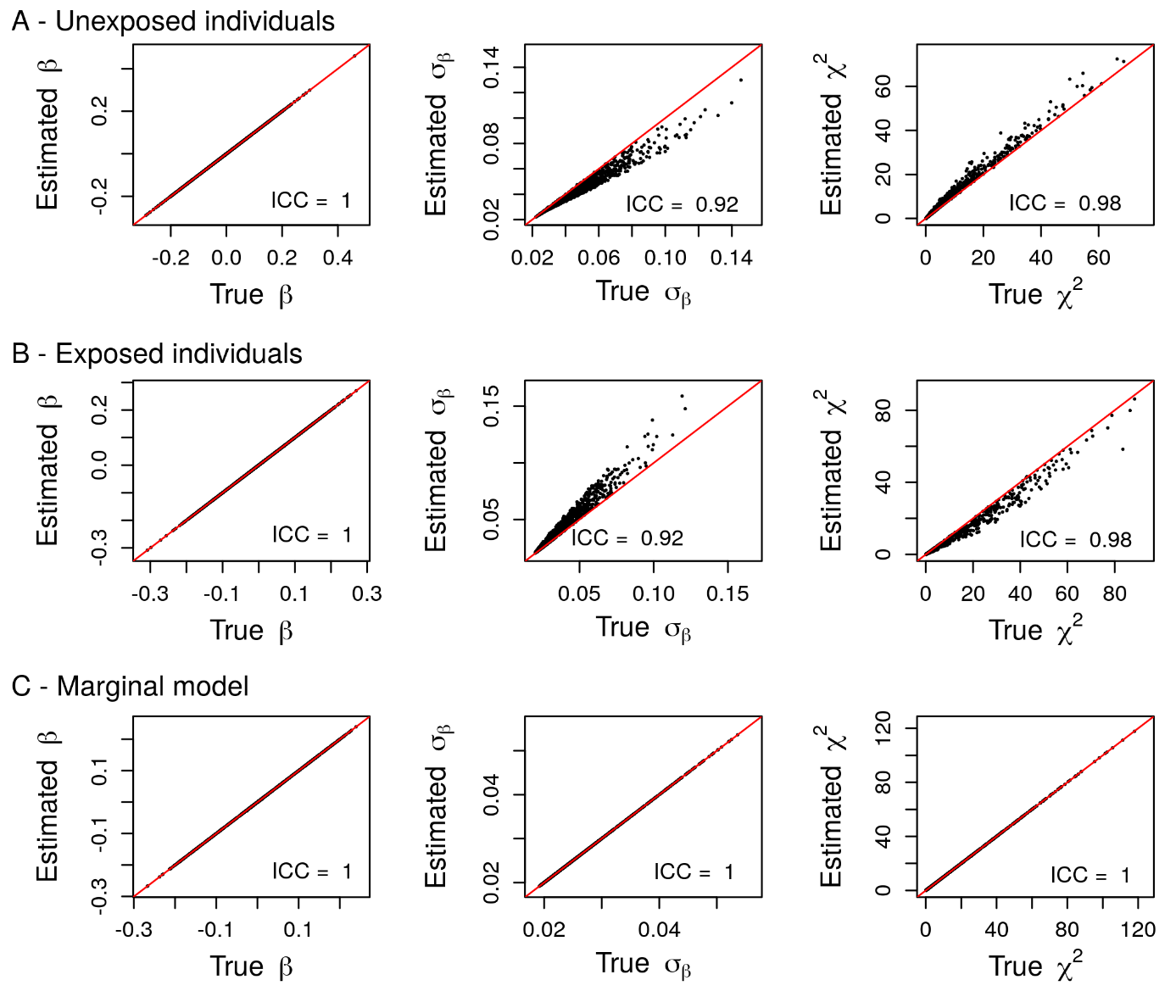


Figure S5. Comparison of the Type I error rate evaluated between summary obtained using individual-level data (blue) and summary statistics estimated using the pipeline (orange) with respect to the different quintiles of the different sources of bias: G-E correlation(A), misspecification of the proportion of exposed individuals (B) and different phenotypic variance in the strata of the exposure (C). Type I error rate were evaluated for the marginal model in all individuals (left), in unexposed individuals only (middle) and in exposed individuals only (right). The dashed line represents the nominal significance threshold (5%).

**
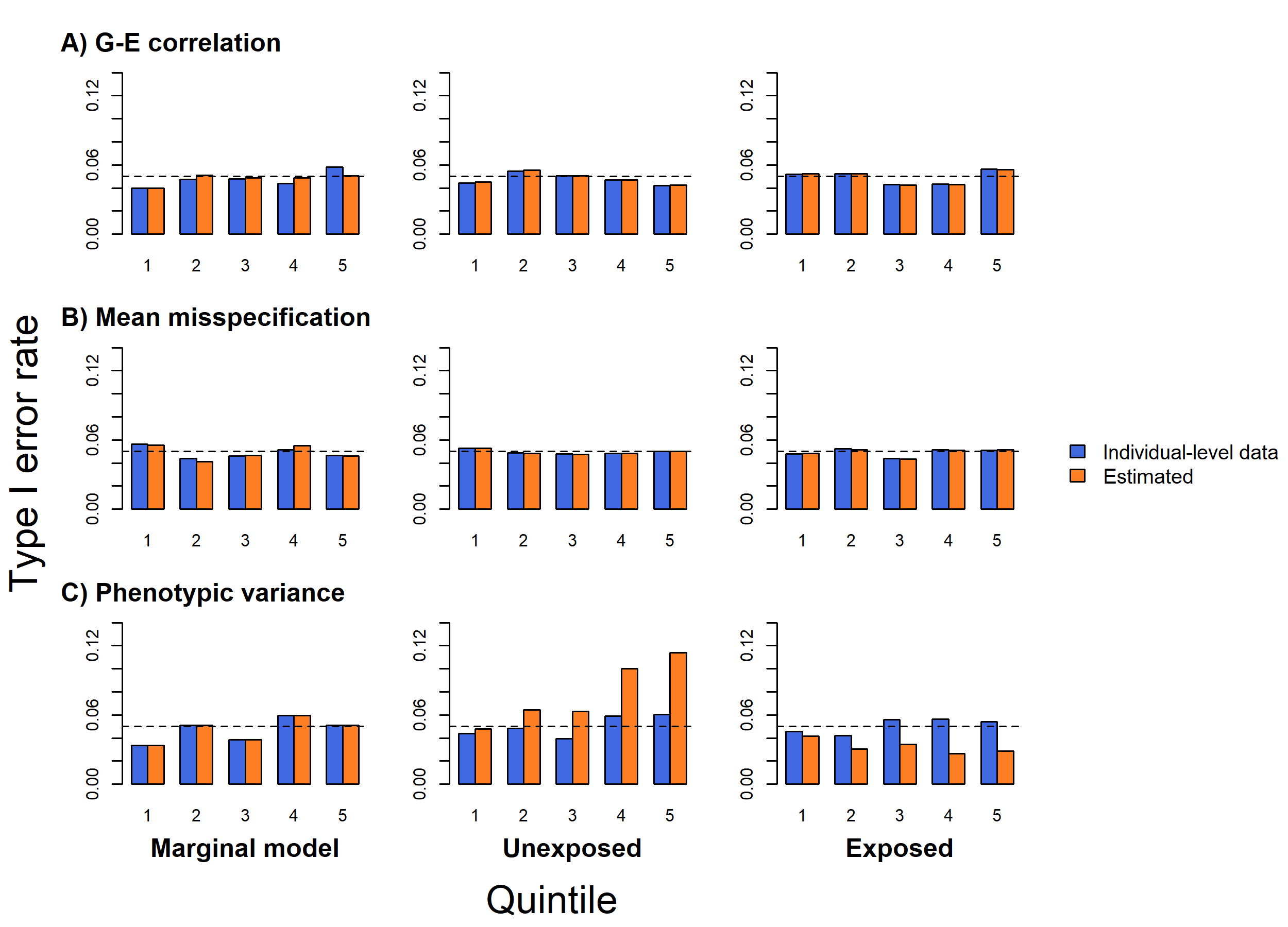
**

Figure S6. Proportion of SNPs with discordant significance results between summary statistics obtained using individual-level data and summary statistics estimated using our pipeline with respect to the different quintiles of the different sources of bias: G-E correlation(A), misspecification of the proportion of exposed individuals (B) and different phenotypic variance in the strata of the exposure (C). Type I error rate were evaluated for the marginal model in all individuals (left), in unexposed individuals only (middle) and in exposed individuals only (right).

**
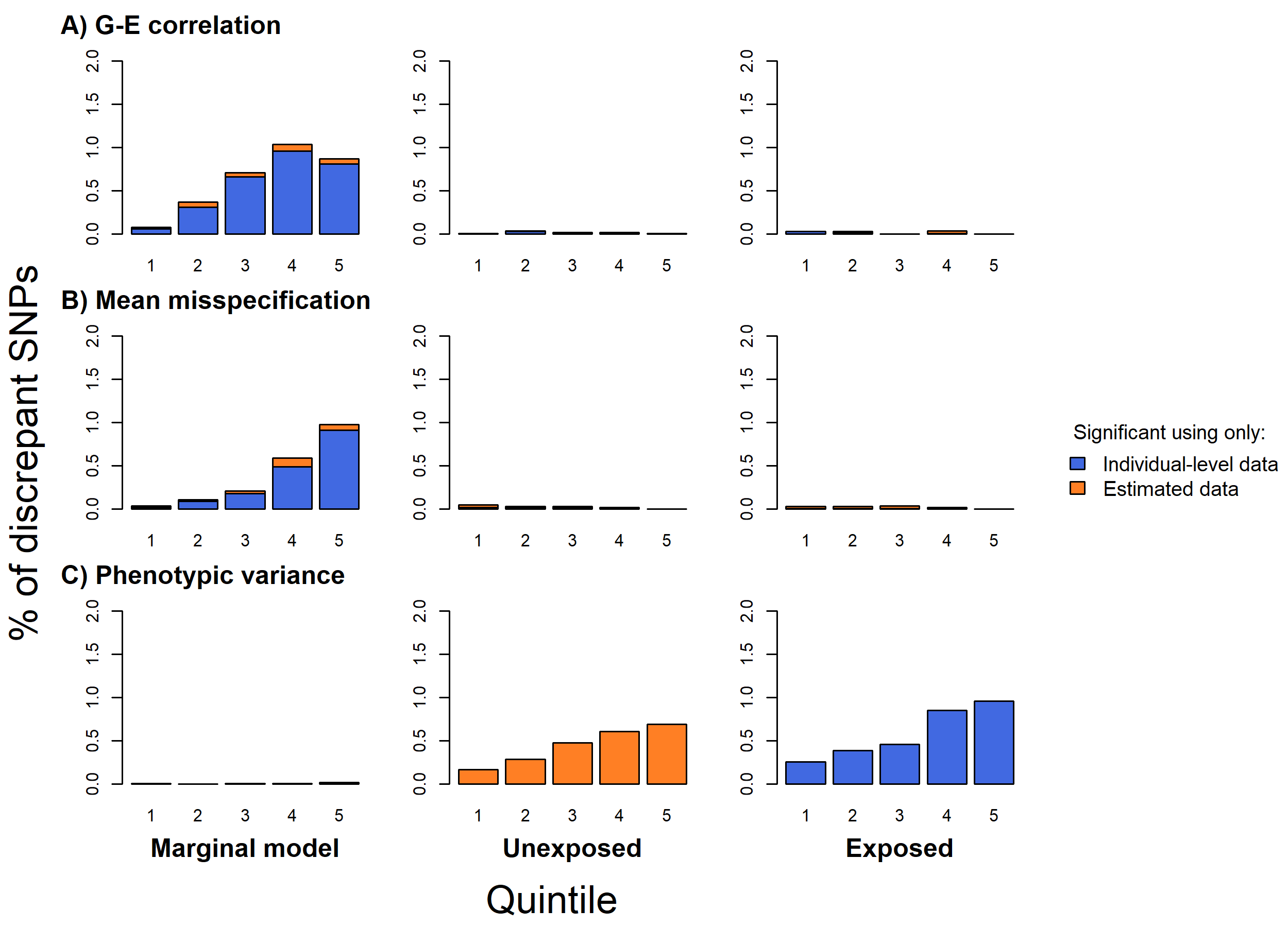
**
